# Expression of BAFF and BR3 in patients with systemic lupus erythematosus

**DOI:** 10.1590/1414-431X20154853

**Published:** 2016-02-02

**Authors:** J.H. Duan, Y. Jiang, H. Mu, Z.Q. Tang

**Affiliations:** Tianjin First Central Hospital, Tianjin, China

**Keywords:** Systemic lupus erythematosus, Peripheral blood mononuclear cells, B cell-activating factor, BAFF receptor

## Abstract

The objective of this study was to examine the relationship between the expression of B cell activating factor (BAFF) and BAFF receptor in patients with disease activity of systemic lupus erythematosus (SLE). Real-time RT-PCR was used to examine BAFF mRNA expression in peripheral blood monocytes of active and stable SLE patients and healthy controls. The percentage of BAFF receptor 3 (BR3) on B lymphocytes was measured by flow cytometry. Soluble BAFF levels in serum were assayed by ELISA. Microalbumin levels were assayed by an automatic immune analysis machine. BAFF mRNA and soluble BAFF levels were highest in the active SLE group, followed by the stable SLE group, and controls (P<0.01). The percentage of BR3 on B lymphocytes was downregulated in the active SLE group compared with the stable SLE group and controls (P<0.01). BAFF mRNA levels and soluble BAFF levels were higher in patients who were positive for proteinuria than in those who were negative (P<0.01). The percentage of BR3 on B lymphocytes was lower in patients who were positive for proteinuria than in those who were negative (P<0.01). The BAFF/BR3 axis may be over-activated in SLE patients. BAFF and BR3 levels may be useful parameters for evaluating treatment.

## Introduction

Systemic lupus erythematosus (SLE) is a prototypic systemic autoimmune disease. In SLE, the loss of tolerance to nucleic acids and their binding proteins results in the generation of autoantibodies that initiate tissue-damaging inflammation (1,2). Recent clinical and experimental studies have indicated that B cells play important roles in the pathogenesis of disease, and this contributes to the development of SLE by presenting antigens and producing cytokines. B cell activating factor (BAFF) belongs to the tumor necrosis factor (TNF) family and is also known as B-lymphocyte stimulator (BLyS), TNF and ApoL-related leukocyte-expressed ligand-1 (TALL-1), TNF homologue that activates apoptosis, nuclear factor-κB, and c-Jun NH2-terminal kinase (THANK), and TNF superfamily member 13B (TNFSF 13B). BAFF is released by myeloid cells, such as monocytes, macrophages, dendritic cells, and neutrophils (3,4). BAFF can bind to its specific receptor, BAFF receptor 3 (BR3, BAFF-R). Through binding to its receptors, BAFF exerts strong stimulation on B cells, and plays a central role in promoting B cell survival and maturation (5,6). Transgenic mice that overexpress BAFF develop autoimmune disease resembling human lupus (7). In this study, we analyzed BAFF mRNA, soluble BAFF, and BR3 in 75 SLE patients. By investigating the associations of BAFF and its receptors with clinical characteristics, we attempted to determine the status and actions of BAFF in SLE, identify new biomarkers for disease activity, and provide evidence for target therapy.

## Material and Methods

### Patients and healthy controls

A total of 75 patients fulfilling the revised SLE classification criteria of the American College of Rheumatology were recruited. The median age of the patients at baseline was 32 years (18-52 years) and the median disease duration was 9 years (4-15 years). Ten patients were male and the other 65 patients were female. The SLEDAI (systemic lupus erythematosus disease activity index)-2000 criteria were used to evaluate disease activity. SLEDAI ≤6 was classified as inactive SLE and SLEDAI >6 was classified as active SLE. Forty active SLE patients were administered oral prednisone ≥1 mg·kg^-1^·day^-1^, intravenous methylprednisolone 40-80 mg/d, or even pulsed methylprednisolone (1 g/day for 3 consecutive days), combined with at least one immunosuppressive drug. Thirty-five inactive SLE patients were treated with oral prednisone ≤0.8 mg·kg^-1^·day^-1^ with or without immunosuppressive drugs. Thirty-five sex- and age-matched healthy volunteers were recruited as healthy controls. The ethics committee of Tianjin First Central Hospital approved this study, and informed consent was obtained from each patient and healthy volunteer.

### Antibodies and reagents

Fluorescein isothiocyanate-conjugated anti-CD19 antibody, PE-conjugated anti-BR3 antibody, and corresponding isotypes were all purchased from BD Pharmingen (USA). The BAFF enzyme-linked immunosorbent assay (ELISA) kit was purchased from R&D Systems (USA). Measurement of 24-h urinary microalbumin levels was performed using a kit from Beckman Coulter (USA).

### Real-time RT-PCR

Total RNA was isolated from peripheral blood mononuclear cells (PBMCs) with TRIzol (Invitrogen, USA) and first-strand cDNA was synthesized by using an RT system (Promega, USA). Quantitative assessment of DNA amplification was detected by the dye SYBR Green by using the Quanti Fast SYBR Green Kit (Qiagen, Germany) according to the manufacturer's instructions. The primers [forward 5'-AAG ACC TAC GCC ATG GGA CAT C-3' and reverse 5'-TCT TGG TAT TGC AAG TTG GAG TTC A-3'] were used to amplify BAFF. Glyceraldehyde-3-phosphate dehydrogenase, which served as the internal reference, was amplified using the following primers: forward 5'-GAA GGT GAA GGT CGG AGT C-3' and reverse 5'-GAA GAT GGT GAT GGG ATT TC-3'. The relative amount of products was determined by the comparative threshold cycle method of 2^-ΔΔCT^. Real-time RT-PCR products were also electrophoresed on 2% agarose gels.

### Flow cytometry

PBMCs were isolated from fresh anti-coagulated blood samples by Ficoll-Hypaque density gradient centrifugation. Cells (1×10^6^ cells/100 µL) were stained with monoclonal antibodies conjugated with PE and fluorescein isothiocyanate. After incubation at 4°C for 30 min in the dark, stained cells were washed with fluorescence-activated cell sorting buffer (1×phosphate-buffered saline, 0.2% fetal bovine serum, 0.09% NAN3). All samples were analyzed on the FACSCanto (BD Biosciences, USA) and data were analyzed with FCS express version 3.0 software.

## ELISA

Serum BAFF levels were detected with ELISA kits according to the manufacturer's instructions.

### Immunoturbidimetric assay

Urinary microalbumin levels were detected on the Beckman Coulter Immage 800. Microalbumin values ≤0.3 mg/24 h were classified as the negative group and those >0.3 mg/24 h were classified as the positive group.

### Statistical analysis

Data are reported as means±SE. Statistical analysis was performed using SPSS 11.5. One-way ANOVA was used for comparison of three or more groups and the Student's Newman-Keuls test was used for comparison of two groups. A P value less than 0.05 was considered to be statistically significant.

## Results

This study was performed on 110 individuals (75 patients and 35 controls). There were no significant differences in age, sex, and body mass index between cases and controls (Table 1).

**Table:**
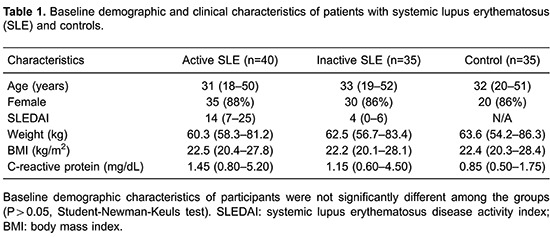

**Figure 1:**
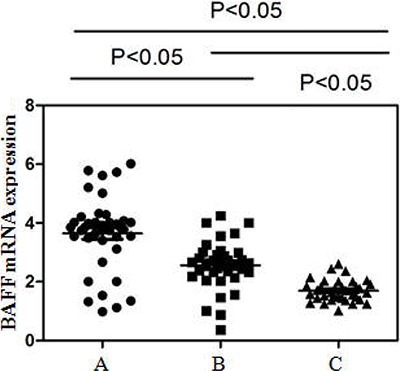
BAFF mRNA expression in PBMCs detected by real-time RT-PCR. *A* , Active systemic lupus erythematosus (SLE); *B* , inactive SLE; *C* , healthy controls. Upregulation of BAFF mRNA expression in PBMCs was 3.92±0.31, 2.58±0.31, and 1.72±0.36, respectively (P<0.05, Student-Newman-Keuls test).

Detectable BAFF mRNA expression in PBMCs was significantly higher in active SLE patients (3.92±0.31) compared with inactive patients (2.58±0.31) and controls (1.72±0.36, both P<0.05, Figure 1). Furthermore, the inactive group had significantly higher BAFF mRNA expression than that of controls (P<0.05).

**Figure 2:**
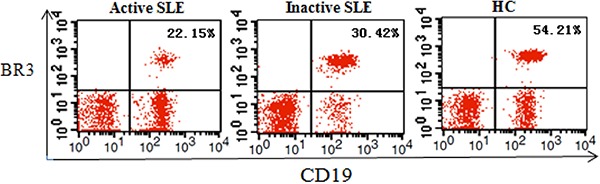
Representative fluorescence-activated cell sorting dot plots of each experimental group. The expression rate of BAFF receptor 3 (BR3) on CD19+ B cells is shown in the three groups. SLE: systemic lupus erythematosus; HC: healthy control.

**Figure 3:**
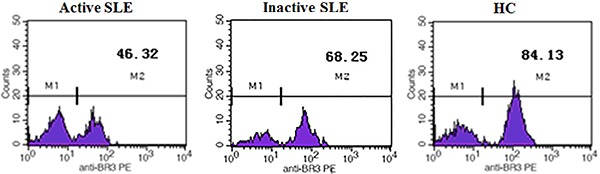
Representative plots of mean fluorescence intensity (MFI) of BAFF receptor 3 (BR3) on CD19+ B cells in the three groups. SLE: systemic lupus erythematosus; HC: healthy control; M1: MFI of negative control; M2: MFI of positive BR3.

Figure 2 shows that BR3 expression was downregulated in SLE. Figure 3 shows representative plots of mean fluorescence intensity (MFI) of BR3 on CD19+ B cells. The MFI of BR3 in the active SLE group (49.77±4.57) and the inactive group (67.96±5.56) was significantly lower than that in controls (85.79±2.09, both P<0.05). MFI of BR3 on CD19+ B cells in the active SLE group was significantly lower than that in the inactive group (P<0.05).

Serum BAFF levels in the active SLE group (3367.22±512.39) were significantly higher than those in the inactive SLE group (2055.37±282.11) and controls (899.70±63.41, both P<0.05).

For active SLE patients, soluble BAFF levels in serum were positively correlated with BAFF mRNA (γ=0.749, P<0.001), and were negatively correlated with BR3 on CD19+ B cells (γ=-0.455, P<0.003).

Renal disease is a known risk factor for poor prognosis in patients with SLE. We further detected microalbumin levels in urine samples of the patients. In the microalbumin-positive group, BAFF mRNA, BR3 on CD19+ B cells, and soluble BAFF serum levels were 2.62±0.32, 52.95±6.87, and 3168±678.00, whereas in the microalbumin-negative group, they were 1.88±0.44, 69.10±5.17, and 2188±461.65, respectively. There were significant differences in the level of BAFF mRNA, BR3 and soluble BAFF between the microalbumin-positive group and the microalbumin-negative group (P<0.05).

## Discussion

SLE is a complex autoimmune disease with considerable heterogeneity in clinical manifestations and disease course (8,9). Despite recent advances in understanding the pathogenesis of SLE, understanding of immunological pathways that are associated with the phenotype in this disease is poor. In the current study, we analyzed BAFF mRNA in PBMCs, BR3 on CD19+ B cells, and serum BAFF levels. We found that the levels of BAFF mRNA and BR3 may be useful biomarkers for measures of SLE activity.

BAFF functions as a potent stimulator to promote maturation and differentiation of B cells, as well as to support survival of B cells and plasmablasts (10,11). Many studies of potential biomarkers have failed to yield evidence of useful correlations with composite measures of disease activity (12). Murine studies have suggested that different manifestations of SLE may be determined by different immunological mechanisms (13). Overexpression of BAFF has been reported in SLE, and antagonists of BAFF activity were investigated in early clinical trials. However, not all SLE patients have increased BAFF in serum, and the relationships of BAFF with clinical manifestations of SLE are controversial. In our study, we found higher BAFF mRNA levels and its soluble molecule in the active SLE group than in the inactive group and controls. Elevation in BAFF levels was more pronounced in the active group than in the inactive group. Elevated BAFF levels after strict therapy are generally accompanied by abnormal clinical consequences, such as higher titers of anti-DNA antibody, lower C3 levels, or newly occurred organ damage, indicating deteriorated active disease or refractory disease. BAFF mRNA and protein levels are highest in DLE+/SLE+ blood, followed by DLE +/SLE-, psoriasis, and normal blood (14). BAFF can exist in multiple forms, such as glycosylated vs unglycosylated, spliced vs unspliced, monomer vs trimer, or polymeric with A proliferation-inducing ligand. Bioactivities of these molecules are not equal, and they cannot all be detected by one antibody kit (15,16).

BR3 is the specific and main receptor of BAFF. More than 90% of B cells in healthy control express BR3 on their surface (17,18). As well as cellular proliferation of lymphocytes, constitutive BAFF/BR3 signaling is critical in NF-κB-inducing kinase-induced nuclear factor-κB pathway activation and survival mechanism in DLBCL cells (19 20 21). Previous studies have demonstrated that BR3 receptor activation promotes normal and malignant B cell survival through both alternative and canonical nuclear factor-κB pathway activation. However, the mechanism of BR3 receptor-induced nuclear factor-κB pathway activation has not been compared between normal and neoplastic human B-lymphoid cells. Our study showed that the proportion of BR3 in lupus B cells was downregulated in SLE patients compared with controls. We thought that downregulation of BR3 in SLE patients may be relevant to uncontrolled active disease or refractory disease. Carter et al. (22) reported that total surface BR3 was identical between SLE patients and healthy controls, but high BAFF levels resulted in occupation of more BR3, and then detectable BR3 was decreased. SLE patients who do not have elevated plasma BAFF levels, but show low detectable BR3 protein in B cells, may still have over-activation of the BAFF-BR3 axis. Chong et al. (14) reported that BAFF-R is elevated in discoid lupus erythematosus skin. A possible explanation for this diversity in results is high levels of inflammatory cells in the skin of DLE patients. Exogenous sources, such as a virus, may lead to increased BAFF production where the dsRNA complex can induce dendritic and salivary gland epithelial cells to enhance BAFF production. However, we detected BR3 protein on the surface of CD19+ B cells in blood in our study. Interaction of BAFF with BR3 stimulates the classic nuclear factor-κB pathway, enhancing B cell survival, growth, and metabolic fitness.

Our study suggests that BAFF and BR3 are useful markers for determination of SLE activity or specific organ involvement. Targeted therapies against BAFF, such as belimumab, will be available for clinical practice in the near future.
